# Flow cytometric significance of cellular alkaline phosphatase activity in acute myeloid leukemia

**DOI:** 10.18632/oncotarget.27356

**Published:** 2019-12-10

**Authors:** Laura G. Rico, Jordi Juncà, Michael D. Ward, Jolene A. Bradford, Jordi Petriz

**Affiliations:** ^1^Functional Cytomics Group, Institut de Recerca contra la Leucèmia Josep Carreras, IJC Campus ICO-Germans Trias i Pujol, Institut Germans Trias i Pujol (IGTP), Universitat Autònoma de Barcelona, UAB, Badalona, Spain; ^2^Institut Català d’Oncologia, Hospital Germans Trias i Pujol (HGTiP), Badalona, Spain; ^3^Thermo Fisher Scientific, Eugene, Oregon, USA

**Keywords:** alkaline phosphatase, acute myeloid leukemia, stem cells, leukemic stem cells, CD34

## Abstract

In this prospective hospital-based cohort study that included 43 newly diagnosed patients with acute myeloid leukemia, flow cytometric cellular alkaline phosphatase (ALP) activity within primitive leukemic cells allowed us to identify two groups of patients at diagnosis according to the numbers of leukemic blasts expressing ≥ 12% of ALP+ cells (27 patients, Group A) and less than 12% of ALP+ cells (16 patients, Group B). Differences in outcome for complete response, relapse or treatment resistance, and exitus were statistically analyzed and were significant, when comparing the two groups. The overall survival (OS) and event-free survival (EFS) differences between Group A and B were statistically significant. The survival of Group A patients was significantly shorter than those for Group B. No significant relationship was detected in outcome when comparing ELN prognostic-risk group based on cytogenetic and molecular profile (patients in the favorable, intermediate, and adverse risk groups). Flow cytometric cellular ALP activity at diagnosis may be used to estimate relapses and disease persistence more accurately. The limitations of our study include the small number of patients enrolled and a short follow-up, due to its prospective nature.

## INTRODUCTION

Flow cytometry immunophenotyping [1] has become one of the mainstream applications for the diagnosis and classification of several hematologic neoplasms. This technology is indispensable for detection of leukemic blasts at a single cell level, clonal lineage assignment, identification of aberrant expression of antigens, and detection of abnormal rare populations of blasts from normal progenitors, with extreme importance in tailoring decision-making. Moreover, functional flow cytometry testing can effectively provide new insights for research and evaluation of disease, and a more complete understanding of the complexities and challenges in the analysis of leukemic stem cells.

Acute myeloid leukemia (AML) arise from stem cells, causing a rapid overproduction of abnormal myeloid blasts, usually requiring immediate treatment. AML is not a single disease and comprises a heterogeneous group of clonal hematopoietic malignancies with poor prognosis, being the most common type of acute leukemia in adults [2–4]. Patients with AML have high overall mortality rate at three years, being higher among patients 65 years or older [5]. Current strategies for the treatment of this disease, assign prognostically favorable subgroups into standard chemotherapy regimens, while unfavorable risk patients are generally considered for undergoing consolidation with allogeneic stem cell transplantation (ASCT).

Alkaline phosphatase (ALP) is expressed at very low levels in somatic cells, whereas it is highly expressed in primitive stem cells. ALP enzyme has a dimeric structure capable of binding Zn^2+^ and Mg^2+^ ions at different sites to stimulate or inhibit its catalytic reaction. In humans, four forms of ALP cDNA have been cloned: one of them is widely distributed (liver, bone, kidney) [6, 7], one restricted to the intestine [8], one to the placenta [9], and one restricted to teratomas and germ cells [10]. ALP hydrolyzes phosphate groups and its activity is involved in a broad range of essential physiological and pathological processes. Immunohistochemical expression of ALP has been reported for embryonal carcinoma and teratoma tumors with two different alkaline phosphatases localized in stem cell populations, as well as in embryonic ectodermal cells [10]. Pluripotent stem cells, embryonic stem cells, induced pluripotent stem cells and embryonic germ cells express highly elevated ALP [11], as demonstrated by western blot, ELISA, immunohistochemistry, and highly sensitive fluorescent and chemiluminescent substrates [12].

Because stem cells lack specific cell surface markers, the identification of this compartment can be difficult, making cancer stem cells especially elusive [13]. Thus, we undertook a prospective cohort study based on our previous work [14], with the objective to investigate whether there might be differences in ALP activity within primitive leukemic cells, and its association with the potential risk of recurrence and mortality in newly diagnosed patients with AML.

## RESULTS

### Patient characteristics and outcomes

**Figure 1:**
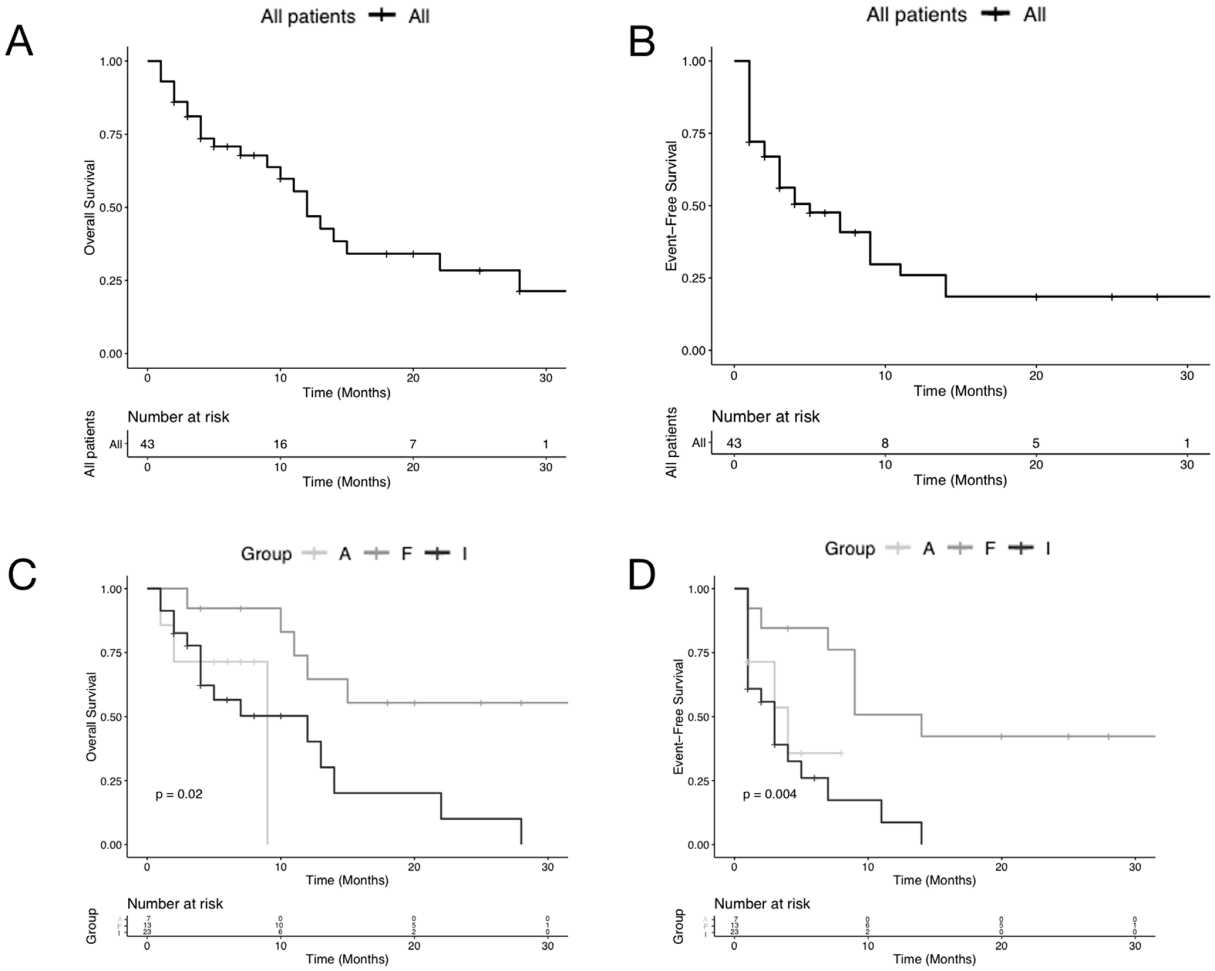
Plots of Kaplan-Meier limit estimates of overall survival and event-free survival curve analysis of acute myeloid leukemia patients at diagnosis. Plots of Kaplan-Meier limit estimates of overall and event-free survival of acute myeloid leukemia ungrouped and grouped patients according to the risk. Plots of Kaplan-Meier limit estimates of overall and event-free survival of acute myeloid leukemia ungrouped patients are shown in (**A** and **B**) respectively. Overall and event-free survival differences for adverse-, intermediate- and favorable-risk patients (A, I, and F) are shown in (**C** and **D**) respectively.

Between May 2015 and May 2018, a total of 106 patients were diagnosed with acute myeloid leukemia at our unit. After exclusion criteria were applied, 43 eligible patients were identified and included in the overall analysis. The characteristics of patients who were included are shown in Supplementary Table 1. The median age of patients was 67 years (range, 26–91 years). Of these, 20 (46.5%) of patients were over 70 years of age, and 23 (53.5%) were under 70 years of age. Overall, 30 (69.8%) patients were male, and 13 (30.2%) were female (Table 1). Median follow-up for OS and EFS was approximately 12 months. The 30-month OS and EFS of all AML patients was 21.3% and 19% respectively (Figure 1A, 1B). The OS and EFS differences for adverse-, intermediate- and favorable-risk patients were also compared (Figure 1C, 1D).

**Table 1:** Numbers of ALP+ blast cells at diagnosis according to patient characteristics

	Patients (*n* = 43), *n* (%)	APL+ blast cells, median (range), %
**Age at diagnosis**		
<70 years	23 (53.5)	13.83 (1.00 - 96.63)
>70 years	20 (46.5)	19.86 (0.26 - 96.63)
**Sex**		
- Male	30 (69.8)	17.36 (0.26 - 96.63)
- Female	13 (30.2)	20.75 (1.62 - 35.91)
**Type of AML**		
- *de novo*	30 (69.8)	18.11 (1.20 - 96.63)
- secondary	13 (30.2)	20.75 (1.00 - 95.92)
**WHO 2017 classification**		
*- AML with recurrent genetic abnormalities*		
- AML with t(8;21)(q22;q22.1); *RUNX1-RUNX1T1*	2 (4.7)	49.17 (1.71 - 96.63)
- AML with inv(16)(p13.1q22) or t(16;16)(p13.1;q22); *CBFB-MYH11*	2 (4.7)	21.81 (13.83 - 29.80)
- AML with mutated *NPM1*	8 (18.6)	13.85 (1.00 - 24.71)
- AML with biallelic mutations of *CEBPA*	3 (7.0)	17.09 (6.53 - 21.75)
*- AML with myelodysplasia related changes*	4 (9.3)	19.19 (9.07 - 95.92)
*- Therapy-related AML*	1 (2.3)	54.95 (54.95 - 54.95)
*- AML not otherwise specified*		
- AML with minimal differentiation	3 (7.0)	15.49 (8.10 - 20.84)
- AML without maturation	3 (7.0)	22.89 (2.65 - 23.76)
- AML with maturation	8 (18.6)	17.43 (0.26 - 37.77)
- Acute myelomonocytic leukemia	4 (9.3)	29.10 (4.96 - 96.63)
- Acute monoblastic/monocytic leukemia	5 (11.6)	18.33 (1.20 - 26.91)
**Cytogenetic alterations**		
- Normal karyotype	20 (46.5)	13.45 (0.26 - 35.27)
- inv(16)(p13.1;q22)	2 (4.7)	21.81 (13.83 - 29.80)
- Monosomy 7 or del 7q	3 (7.0)	23.76 (15.49 - 54.95)
- Trisomy 8	5 (11.6)	35.91 (26.03 - 96.63)
- t(8;21)(q22;q22.1)	2 (4.7)	49.17 (1.71 - 96.63)
- t(11q23)	1 (2.3)	2.65 (2.65 - 2.65)
- Complex karyotype	3 (7.0)	9.53 (4.96 - 20.84)
- Others	7 (16.3)	20.75 (8.10 - 95.92)
**Molecular alterations**		
- *NPM1* mutated without *FLT3-*ITD	5 (11.6)	5.35 (1.00 - 17.89)
- *FLT3-*ITD with or without *NPM1* mutated	5 (11.6)	18.97 (1.20 - 26.03)
- *FLT3*-TKD	1 (2.3)	29.8 (29.8 - 29.8)
- *CEBPA* mutated	3 (7.0)	17.09 (6.53 - 21.75)
- c-Kit mutated	1 (2.3)	13.83 (13.83 - 13.83)
- Wild type	28 (65.1)	21.56 (0.26 - 96.63)
**Cytogenetic and molecular prognostic-risk group**		
- Favorable	13 (30.2)	13.83 (1.00 - 96.63)
- Intermediate	23 (53.5)	22.89 (0.26 - 96.63)
- Adverse	7 (16.3)	9.53 (1.62 - 26.03)
**Relevant LSC markers**		
- CD34+/CD123+/CD117+	28 (65.1)	20.79 (0.26 - 96.63)
- CD34+/CD123-/CD117+	3 (7.0)	95.92 (2.65 - 96.63)
- CD34-/CD123+/CD117+	9 (20.9)	9.82 (1.00 - 30.39)
- CD34-/CD123+/CD117-	3 (7.0)	18.33 (11.98 - 26.91)
**Initial treatment**		
- CETLAM12<70^*^	20 (46.5)	11.82 (1.00 - 96.63)
- CETLAM12>70^†^	5 (11.6)	15.49 (0.26 - 35.27)
- FLUGAZA clinical assay^‡^	11 (25.6)	21.75 (17.64 - 96.63)
- Others^§^	7 (16.3)	11.98 (1.62 - 30.39)
**Post remission therapy**		
- Allogeneic SCT	12 (28.0)	23.02 (1.00 - 96.63)
- Other	31 (72.0)	17.89 (0.26 - 96.63)

Abbreviations: APL, alkaline phosphatase; AML, acute myeloid leukemia; WHO, world health organization; NPM1, nucleophosmin 1; FLT3-ITD, fms-like tyrosine kinase 3 internal tandem duplication; FLT3-TKD, fms-like tyrosine kinase 3 tyrosine kinase domain; SCT, stem cell transplant.

^*^Cytarabine + idarubicin; ^†^Cytarabine + fludarabine; ^‡^Cytarabine + fludarabine / Azacitidine; ^§^Azacitidine / Decitabine / Cytarabine + idarubicin + quizatinib or placebo / None.

### Alkaline phosphatase activity differences in AML: identification of two patient groups

**Figure 2:**
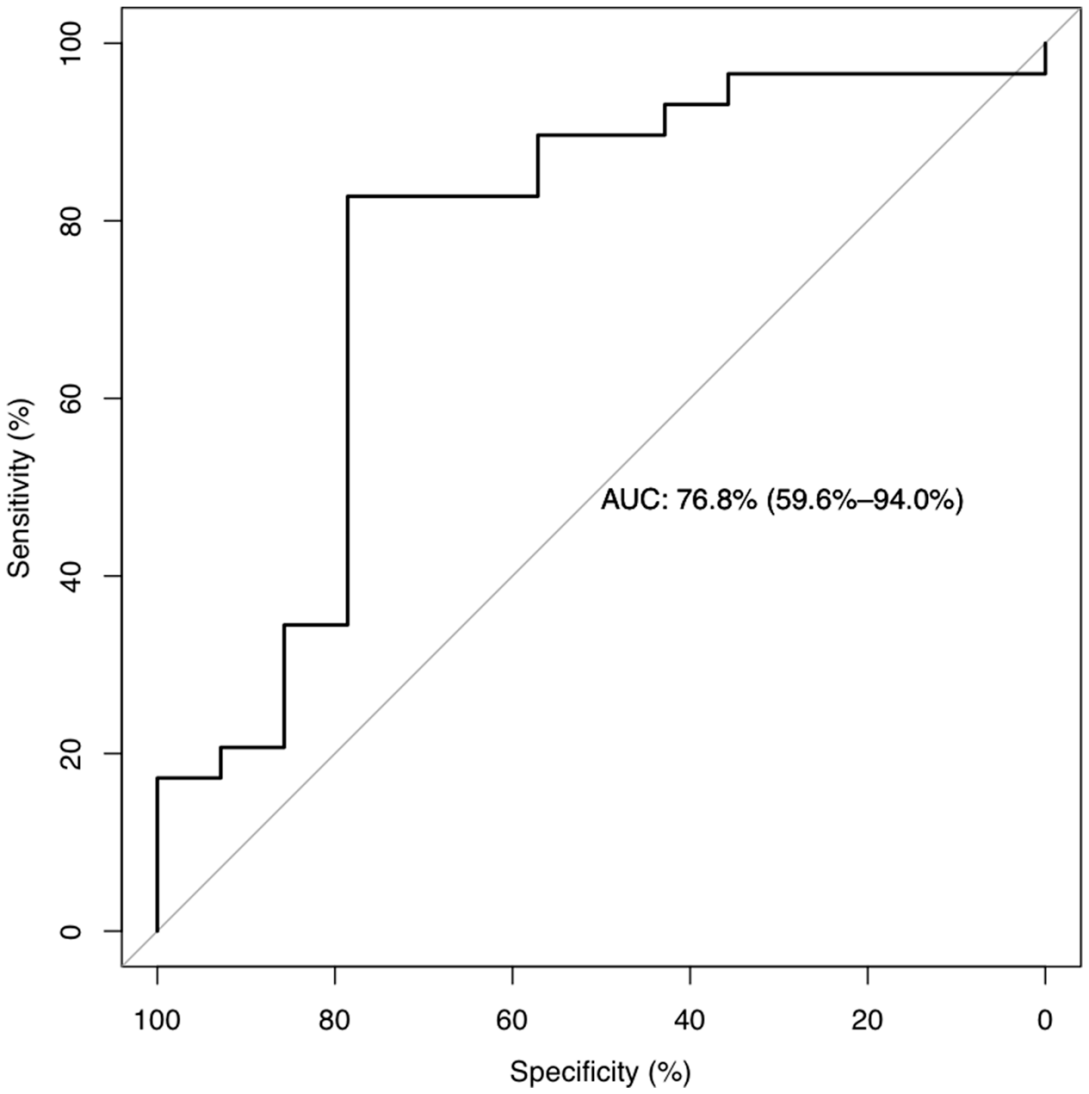
Receiver operating characteristic (ROC) curves. According to ROC curve analysis, 12% of ALP+ was confirmed as the cut-off point of ALP+ leukemic cell counting for survival outcome of AML patients. ROC curve analysis (area under the curve = 0.768, 95% CI = 0.596 to 0.94, *P*-value < 0.0001) classified two identifiable ALP groups of patients at diagnosis according to the numbers of leukemic blasts expressing ≥ 12% of ALP+ cells (27 patients in Group A) and less than 12% of ALP+ cells (16 patients in Group B).

Differences in ALP+ blast cells at diagnosis were analyzed for the entire population. The value of ALP+ blast cells was expressed as percentages of the median and ranges around the median. Table 1 summarizes the median and range of ALP+ blast cells at diagnosis according to the age, sex, type of AML (*de novo* or secondary), WHO 2017 classification [15], cytogenetic and molecular alterations, European LeukemiaNet (ELN) prognostic-risk group based on cytogenetic and molecular profile [16], relevant blast immunophenotyping (CD34/CD117/CD123 backbone), initial treatment, and post-remission therapy. The diagnostic performance of the ALP test as a binary classifier system, or the accuracy of the method to discriminate two ALP+ populations, was used to determine its predictive value. As shown in Figure 2, Receiver Operating Characteristic (ROC) curve analysis (area under the curve (AUC) = 0.768, 95% Confidence Interval (CI) = 0.596 to 0.94, *P*-value < 0.0001) allowed us to classify two identifiable ALP groups of patients at diagnosis according to the numbers of leukemic blasts expressing ≥ 12% of ALP+ cells (27 patients in Group A) and less than 12% of ALP+ cells (16 patients in Group B).

### Differences in outcomes between groups

Overall, 43 patients, with a median age of 63 years (range 26–91), had a median value of 18.33% ALP+ blast cells (range 0.26–96.93). Twenty-seven patients in Group A had a median value of 23.76% of ALP+ blast cells ranging from 13.83 to 96.63), and 16 patients in Group B had a median value of 4.99% of ALP+ blast cells ranging from 0.26 to 11.98 (*P*-value < 0.0001, 95% CI: 15.79 – 24.84).

Differences in outcome comparing the two groups were also analyzed. Seven patients in Group A achieved a complete response (25.9%), in contrast with 11 patients (68.8%) in Group B (*P*-value = 0.01, 95% CI = 1.34 to 30.99). Twenty-five patients in Group A relapsed or showed treatment resistance (92.6%), whereas this occurred only in 5 patients (31.3%) in Group B (*P*-value = <0.0001, 95% CI = 0.00 to 0.26). Twenty-two patients in Group A died (81.5%), whereas 5 died in Group B (31.3%) (*P*-value = 0.002, 95% CI = 0.02 to 0.52). Table 2 summarizes the differences between Group A (≥12% ALP+ blasts cells) and Group B (<12% ALP+ blasts cells) adjusted for the age, sex, type of AML (*de novo* or secondary), WHO Classification of AML (2017 edition), cytogenetic and molecular alterations, prognostic-risk group based on cytogenetic and molecular profile, relevant blast immunophenotyping (CD34/CD117/CD123 backbone), post-remission therapy, and outcomes (complete response achievement, relapse or treatment resistance, and exitus).

**Table 2:** Differences between Group A and Group B regarding total number of ALP+ blast cells at diagnosis

	Overall (*n* = 43)	Group A (*n* = 27)	Group B (*n* = 16)	*P*-value (95% CI)
ALP+ blast cells, median (range), %	18.33 (0.26 - 96.63)	23.76 (13.83 - 96.63)	4.99 (0.26 - 11.98)	<0.0001 (15.79 - 24.84)^****^
Age at diagnosis, median (range), years	63.0 (26 - 91)	73.0 (26 - 81)	59.5 (31 - 91)	0.38 (-5.00 - 17.00)
**Sex, *n* (%)**				
- Male	30 (69.8)	17 (63.0)	13 (81.3)	0.31 (0.50 - 16.99)
- Female	13 (30.2)	10 (37.0)	3 (18.7)	
**Type of AML, *n* (%)**				
- *de novo*	30 (69.8)	19 (70.4)	11 (68.8%)	1 (0.22 - 4.93)
- secondary	13 (30.2)	8 (29.6)	5 (31.2%)	
**WHO 2017 classification, *n* (%)**				
- AML with recurrent genetic abnormalities	15 (34.9)	9 (33.3)	6 (37.5)	1 (0.27 - 5.18)
- AML with myelodysplasia related changes	4 (9.3)	3 (11.1)	1 (6.3)	1 (0.00 - 7.47)
- Therapy-related AML	1 (2.3)	1 (3.7)	0 (0.0)	1 (0.00 - 65.75)
- AML not otherwise specified	23 (53.5)	14 (51.9)	9 (56.2)	1 (0.29 - 4.99)
**Molecular alterations, *n* (%)**				
- *NPM1* mutated without *FLT3-*ITD	5 (11.6)	1 (3.7)	4 (25.0)	0.06 (0.71 - 441.52)
- *FLT3-*ITD with or without *NPM1* mutated	5 (11.6)	3 (11.1)	2 (12.5)	1 (0.09 - 11.26)
- *NPM1* and *FLT3* wild type	33 (76.8)	23 (85.2)	10 (62.5)	0.14 (0.05 - 1.58)
**Cytogenetic and molecular prognostic-risk group, *n* (%)**				
- Favorable	13 (30.2)	7 (25.9)	6 (37.5)	0.50 (0.36 - 7.82)
- Intermediate	23 (53.5)	17 (63.0)	6 (37.5)	0.12 (0.08 - 1.50)
- Adverse	7 (16.3)	3 (11.1)	4 (25.0)	0.39 (0.37 - 20.75)
Blasts at diagnosis, median (range), %	45.0 (6.0 - 98.0)	50.0 (18.0 - 98.0)	39.0 (6.0 - 88.8)	0.35 (-7.99 - 23.00)
WBC at diagnosis, median (range), *n* × 10^9^/L	8.5 (0.5 - 249.1)	5.9 (0.7 - 249.1)	13.3 (0.5 - 65.4)	0.93 (-10.80 - 6.60)
**Relevant LSC markers, *n* (%)**				
- CD34+/CD123+/CD117+	28 (65.1)	19 (70.0)	9 (56.2)	0.51 (0.12 - 2.39)
- CD34+/CD123-/CD117+	3 (7.0)	2 (7.5)	1 (6.3)	1 (0.01 - 17.38)
- CD34-/CD123+/CD117+	9 (20.9)	4 (15.0)	5 (31.2)	0.26 (0.45 - 15.67)
- CD34-/CD123+/CD117-	3 (7.0)	2 (7.5)	1 (6.3)	1 (0.01 - 17.38)
**Post remission therapy, *n* (%)**				
- Allogeneic SCT	12 (28.0)	8 (30.0)	4 (25.0)	1.0000 (0.26 - 7.00)
- Other	31 (72.0)	19 (70.0)	12 (75.0)	
**Outcomes, *n* (%)**				
- Complete response	18 (41.9)	7 (25.9)	11 (68.8)	0.01 (1.34 - 30.99)^*^
- Relapse or Treatment resistance	30 (69.8)	25 (92.6)	5 (31.3)	<0.0001 (0.00 - 0.26)^****^
- *Exitus*	27 (62.8)	22 (81.5)	5 (31.3)	0.002 (0.02 - 0.52)^**^

Abbreviations: CI, confidence interval; ALP, alkaline phosphatase; n, number; AML, acute myeloid leukemia; WHO, world health organization; NPM1, nucleophosmin 1; FLT3-ITD, fms-like tyrosine kinase 3 internal tandem duplication; WBC, white blood cells; L, liter; LSC, leukemic stem cell; SCT, stem cell transplant.

^*^ *p*-value < 0.05; ^**^ *p*-value <0.01; ^****^ *p*-value <0.0001.

### Survival differences between groups

The overall survival (OS) and event-free survival (EFS) differences between Group A and B were statistically significant. The survival for Group A patients was significantly shorter than for Group B.

**Figure 3:**
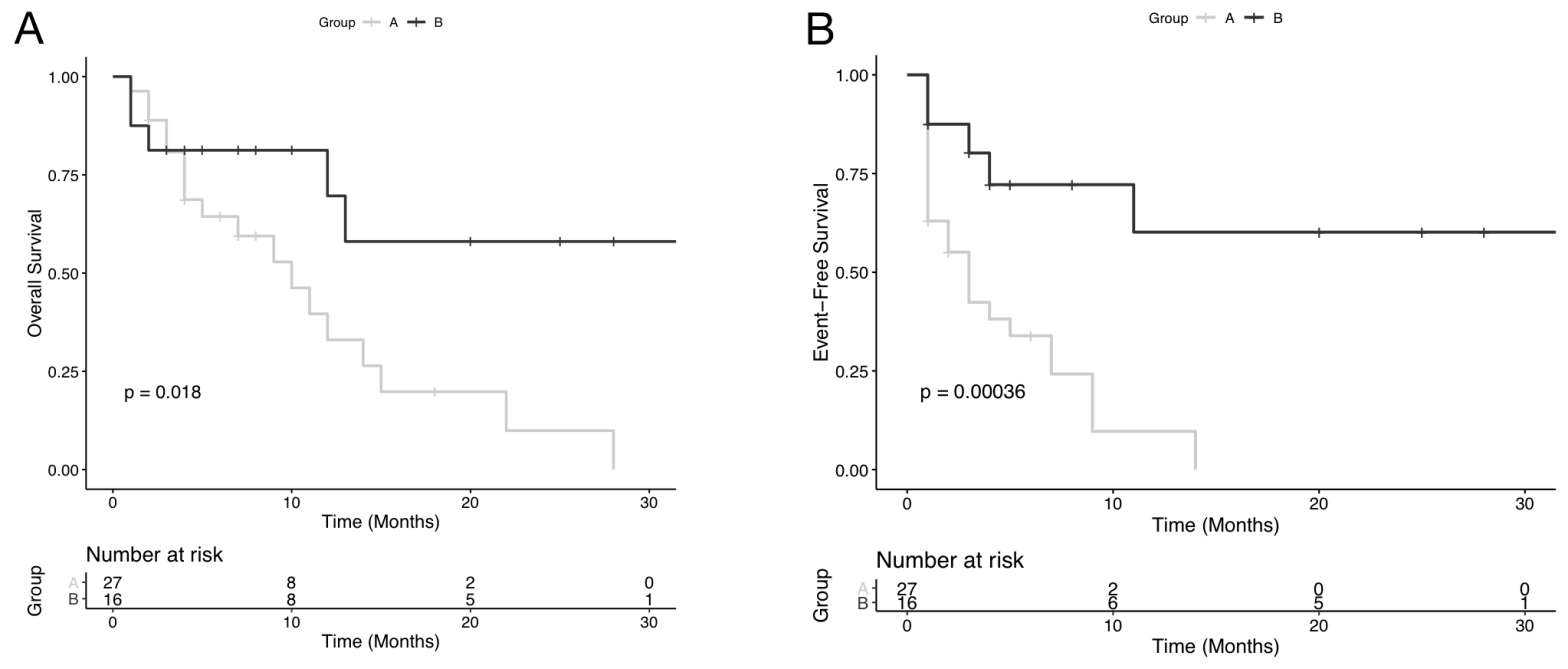
Plots of Kaplan-Meier limit estimates of overall survival and event-free survival curve analysis of acute myeloid leukemia patients, according to the numbers of alkaline phosphatase (ALP) positive leukemic cells at diagnosis. Overall survival after diagnosis was compared between high ALP Group (A) and low ALP Group (B). Patients with ALP+ leukemic cells ≥ 12% achieved shorter OS than those with ALP+ leukemic cells < 12%. According to ROC curve analysis, 12% of ALP+ was confirmed as the cut-off point of ALP+ leukemic cell counting for survival outcome of AML patients. Kaplan-Meier plots of overall survival for grouped acute myeloid leukemia patients are shown in (**A**). Plots of Kaplan-Meier product limit estimates of event-free survival of grouped patients according to the numbers of ALP positive leukemic cells at diagnosis are shown in (**B**).

The 25-month OS in patients with ≥ 12% of ALP+ leukemic cells (Group A) was 9%, and the 10-month EFS in the same group was 10%. The 30-month OS and EFS in patients with <12% of ALP+ leukemic cells (Group B) was 58% and 60% respectively (Figure 3).

### Multivariable analysis

Univariate and multivariate Cox proportional hazards models were used to identify factors that are associated with OS and EFS in patients with AML (Table 3). ALP expression was a significant predictor of OS on univariate (hazard ratio [HR] = 0.32, 95% CI = 0.11 to 0.87, *P*-value < 0.025) analysis. ALP expression was a significant predictor of EFS on univariate ([HR] = 0.19, 95% CI = 0.17 to 0.52, *P*-value = 0.0012) and multivariate ([HR] = 0.25, 95% CI = 0.09 to 0.70, *P*-value < 0.0079) analyses. In the univariate Cox model for OS, age, favorable and intermediate risk, AML type, and ALP Group were associated with a significant impact on survival. The only factor that retained their significance on multivariate analysis was age. In the univariate Cox model for EFS, age, favorable and intermediate risk, and ALP Group were associated with a significant impact on progression/relapse. The only factors that retained their significance on multivariate analysis were favorable risk and ALP group.

**Table 3:** Multivariate analyses

Variable	Overall Survival (OS)				Event-Free Survival (EFS)			
	Univariate Hazard ratio (95% CI)	*P*-value	Multivariate Hazard ratio (95% CI)	*P*-value	Univariate Hazard ratio (95% CI)	*P*-value	Multivariate Hazard ratio (95% CI)	*P*-value
**Age**	1.10 (1.00 – 1.10)	0.0014^**^	1.04 (1.00 – 1.08)	0.0341^*^	1.00 (1.00 –1.10)	0.039^*^	1.01 (0.99 – 1.05)	0.21
**Sex**	1.40 (0.56 – 3.40)	0.48	0.58 (0.24 – 1.92)	0.47	1.00 (0.45 – 2.20)	1.00	-	-
**ALP Group**	0.32 (0.11 – 0.87)	0.025^*^	0.47 (0.15 – 1.34)	0.15	0.19 (0.07 – 0.52)	0.0012^**^	0.25 (0.09 – 0.70)	0.0079^**^
**AML Type**	4.20 (1.80 – 9.80)	0.0009^***^	1.50 (0.49 – 5.27)	0.43	1.60 (0.71 – 3.50)	0.27	0.48 (0.16 – 1.46)	0.20
**Favorable Risk**	0.25 (0.09 – 0.70)	0.0086^**^	0.33 (0.07 – 1.69)	0.18	0.24 (0.09 – 0.61)	0.0026^**^	0.23 (0.06 – 0.93)	0.039^*^
**Intermediate Risk**	2.70 (1.00 – 5.80)	0.042^*^	0.78 (0.19 – 3.11)	0.72	3.10 (1.40 – 6.80)	0.0053^**^	1.01 (0.33 – 3.12)	0.99
**Adverse Risk**	1.60 (0.44 – 5.70)	0.50	-	-	1.20 (0.41 – 3.60)	0.72	-	-
**% Blasts**	0.99 (0.97 – 1.00)	0.20	1.00 (0.98 – 1.03)	0.76	1.00 (0.98 – 1.00)	0.99	-	-
**WBC**	0.99 (0.98 – 1.00)	0.46	1.00 (0.99 – 1.01)	0.94	1.00 (0.99 – 1.00)	0.64	-	-

Abbreviations: CI, confidence interval; ALP, alkaline phosphatase; AML, acute myeloid leukemia; WBC, white blood cells.

^*^ *p*-value < 0.05; ^**^ *p*-value < 0.01; ^***^ *p*-value < 0.001.

## DISCUSSION

Acute myeloid leukemia is a heterogeneous clonal disorder associated with a relatively high early mortality rate and poor overall chances for recovery. Even with current treatments, the relative 5-year survival rate for patients with AML is only 5% after the age of 65 [17]. Current diagnosis and medical treatment of AML is expensive, not just in terms of cost, but also in terms of the impact on prolonged overall treatment duration for patients and their families. Despite new therapy options having been developed to treat AML, advancement in understanding the pathogenesis of AML is continuously needed. Furthermore, the capability to accurately predict survival is often difficult, raising the need for new predictive biomarkers in AML therapeutics.

The fundamental goal of personalized medicine, aimed at providing patient-specific molecular insights, combines genomic, clinical, and other types of data, to provide strategies aimed at treatment optimization based on individual patient characteristics. In recent years, advances in genotypic arrays, digital PCR, and next-generation sequencing, also known as high-throughput sequencing, have played a key role in developing new assays, such as those for predicting AML relapse after ASCT, for mutational screening, risk stratification, guided therapy, or Measurable residual disease (MRD) monitoring. Multiparameter flow cytometry in the clinical laboratory has also become an essential technology to increase the sensitivity of leukemia detection at the single cell level, especially for identifying very low levels of residual disease immediately after treatment and during follow-up.

Assessment of MRD is today considered indispensable for forecasting the significance and association with treatment outcome and survival [18]. However, the sensitivity of the assays, phenotypic shifts at relapse, low frequency of residual leukemic stem cells, quality of the marrow aspirate, or the use of peripheral blood, may lead to identifying false-positive and false-negative patient groups by current approaches [19]. AML comprises a dynamic disease with a very complex landscape of aberrant phenotypic, cytogenetic and molecular genetic abnormalities, that importantly confer independent biological properties of the leukemic cells.

In 1970, Clarkson et al. found leukemic cells to behave as a self-maintaining population [20]. Although their data did not preclude some influx of leukemic “stem cells” from an unrecognized precursor compartment, they found no evidence to support this hypothesis. On the same subject of AML, Bonnet and Dick (1997), demonstrated that leukemic cells obtained from patients possess differentiative and proliferative capacities, as well as the self-renewal properties found in leukemic stem cells [21]. However, the heterogeneity of the stem cell compartment supports that the stem cell concept is not necessarily associated with a specific cellular entity, but rather a function that can be assumed by numerous diverse cell types. As a result, the cancer stem cell concept cannot be universally applicable, based on complex evolution, phenotypic heterogeneity and therapeutic challenges of many human cancers, making difficult the identification of rare molecular and phenotypic-based putative leukemic stem cells [22, 23].

Aside from antigen expression properties on AML bulk cells, and that antigen coexpression on leukemic stem cells (LSC) makes it difficult to specifically target this compartment, LSCs have unique features based on metabolic response to drugs, cell stress control, and other key aspects of stemness and chemotherapy resistance. Based on the clonal nature of tumors, and after the demonstration that leukemic stem cells from patients with AML reconstitute the full spectrum of phenotypes in the malignant populations that they regenerate in transplanted mice, we hypothesized that ALP could help to identify primitive leukemic cells [14]. Moreover, it has been demonstrated that ALP activity may be useful for the diagnosis of acute myelomonocytic and monocytic leukemia in dogs [24]. In parallel with flow cytometric immunophenotyping, Stokol et al. used a cytochemical staining approach for ALP activity, concluding that ALP activity in canine AML can be especially helpful when flow cytometric results are inconclusive. We then decided to implement a new ALP flow cytometric method test, using a novel fluorogenic live cell permeant substrate for ALP [12], since this stain efficiently visualized pluripotent stem cells such as human embryonal carcinoma, murine and human embryonic stem cells and induced pluripotent stem cells under fluorescent microscopy [25]. Our method uses a no-lyse no-wash approach, offering opportunities to combine live cell response and functional assessment in combination with cell immunophenotyping (Supplementary Figure 1), while minimizing sample preparation effects on the cell biology as the primary goal [26]. This method can also be used to identify rare cells [27]. Additionally, assays dealing with live kinetic analysis, like calcium flux and enzyme rate, or assays that depend on continued active drug transport just prior to analysis, like side population analysis must be done with live cells, preferably with as little sample manipulation as possible. Recent work by the authors [14] showed elevated ALP activity in CD34+ cells in highly refractory cancers. This study was made possible by using this new non-toxic cell-permeant fluorescent ALP for live cells without need to add multidrug resistant transporter inhibitors, taking advantage of one of the most-widely used enzyme-based standards, known as the aldehyde dehydrogenase (ALDH) assay [28]. Previously, we demonstrated measurement of ALP activity with ABCG2 efflux pump activity using this reagent in combination with side population analysis, making its use feasible for primitive stem cells expressing ABC transporters. This can only be done with live healthy cells at the point of analysis [14].

Measuring live cell response function and immunophenotype with the minimum possible sample manipulation, may increase the potential for discovery of clinically relevant cell subsets. This area of research is particularly interesting from a both theoretical and practical point of view. As far as we know, some phenotypic characteristics may relate to clinical outcome in AML patients, i.e, CD34 negativity related with NPM1 mutation, CD56 expression in AML with (8;21), or CD2, CD36, CD11b or CD56 positivity in other types of AML. However, attempts to characterize these associations have yielded inconclusive results. Given we lack specific markers to identify if a given phenotype is tightly linked or specifically caused by some mutations, maybe the only way to identify rare cell types is by combining phenotype information with and functional assays.

At the light of our results, increased cellular ALP activity in AML with no evidence of aberrant antigen expression is still unclear and should be confirmed in a large series of patients. Importantly in the low residual disease, MRD evaluation by whatever the method (leukemia-associated immunophenotypes (LAIPs) identification *vs*. “different from normal” approaches) has the value of indicating the clinicians “how things are going”, and that it may have a definitive underlying functional importance is to achieve and maintain a low or negative value for ALP activity, because a marked increase in this biomarker could be interpreted to reflect increased pathological functional activity in a not yet detected leukemic but previously fully identified population, the emergence of a new and probably more immature and aggressive type of cells that may in fact determine the clinical course of the patient, or to provide some clues for more proliferative advantages related to a given phenotype.

In this work, we have shown and validated that ALP expression by blast cells at diagnosis may have a significant impact to identify two different groups of patients. In addition, we have demonstrated that ALP also has a significant impact to predict complete response, relapse or treatment resistance, and exitus, independently of variables such as age, sex, type of AML (*de novo* or secondary), WHO Classification of AML (2017 edition), cytogenetic and molecular alterations, ELN prognostic-risk group based on cytogenetic and molecular profile, relevant blast immunophenotyping (CD34/CD117/CD123 backbone), initial treatment, and post-remission therapy (Table 2).

Despite the cohort’s small number, some risk factors correlated with overall survival and event free survival. Specifically, age entered all univariate and multivariate models except with the EFS in the multivariate, and favorable risk only did not correlated well with OS in the multivariate. Intermediate risk correlated well with OS and EFS in the univariate. Finally, the ALP group was significant in the univariate model for EFS and OS, as well as in the multivariate model for EFS. The ALP group only did not correlate with OS in the multivariate (Table 3).

Surprisingly, the percentage of ALP+ blast cells in the intermediate risk group (median: 22.89, range: 0.26–96.63) was higher than that of the adverse risk group (median: 9.53; range: 1.62–26.03), suggesting that the intermediate risk group may include a relative number of patients with adverse risk (Table 1). Despite that, this result can also be limited by the bias of the cohort’s small number, this observation is supported by the OS and EFS Kaplan-Meier analyses according to favorable, intermediate, and adverse definitions (Figure 1C and 1D). Even with these limitations, our results have significant clinical and scientifically important implications.

The outcome of AML treatment is highly variable and still not individually predictable [29]. Currently, the most refined predictive data that relate to the prognosis of AML are those derived of cytogenetic and molecular analysis. However, other most determinant predictive factors may exist, and that they may reflect the degree of immaturity of the main burden of cells that proliferate in a given case of AML, and that this degree of immaturity (as judged by a functional analysis) is more prognostically determinant that the cytogenetic or molecular characteristics of leukemic cells. However, this is only a hypothesis to verify, and, in fact, we have no explanation for the apparently contradictory result of the lack of correlation of ALP and cytogenetics. Relative to other cancers, AML has a comparatively low level of genetic heterogeneity, suggesting that epigenetic heterogeneity is of primary importance. Thus, epigenetic factors such as DNA methylation, genomic imprinting, histone modifications, and expression control by noncoding RNA, may also play an important role. If confirmed in larger series of patients, our results open a new perspective of research in the analysis of outcome in AML. Moreover, if cellular alkaline phosphatase activity is a prognosticator at diagnosis, it could help to investigate how to reach the ultimate goal of individualized risk assessment as a guide for therapy decision-making, or to design new novel treatment targets in AML. The development of novel ALP inhibitors and modulators [30] for the treatment of AML are promising approaches to reduce the increased mortality associated with this heterogeneous disease characterized by a multitude of molecular abnormalities. Since several methods are only applicable for MRD detection on a clinical routine basis, cellular ALP could help to better classify or to predict the behavior of the disease, as well as to is really predicting the outcome in a given patient. Furthermore, it would be also very interesting to study the comparative value of monitoring MRD regularly with flow cytometric ALP, as truly negative non-detectable MRD status has strong clinical relevance in AML, making it an essential tool in the overall strategy adopted to treat AML.

The limitations of our study include the small number of patients enrolled and a short follow-up, due to its prospective nature. We are fully aware that standard risk assessment markers have been well validated in numerous large studies and suggest that findings in this small set of 43 patients may not be valid in a larger population. Despite this small number, this study offers evidence to the hypothesis that intrinsic cellular ALP activity at diagnosis may be used to estimate relapses and disease persistence more accurately. Larger studies will be needed to determine whether ALP activity in primitive leukemic cells is associated with the potential risk of recurrence and mortality in newly diagnosed patients with AML.

Since the discovery of the hybridoma technology, many monoclonal antibodies have been available for laboratory studies, making possible to dissect human malignancy. In the next few years, more investigations should shed light on the translational utility of new available biological indicators aimed at detecting cell function, and discovering in combination with flow cytometry immunophenotyping, the complex and heterogeneous biology of the stem cell compartment in human pathology.

## MATERIALS AND METHODS

### Study design and patient population

This was a prospective cohort study of patients newly diagnosed with acute myeloid leukemia and completed follow-up. From May 2015 through May 2018, 106 patients were diagnosed with AML at the hematological cytometry unit at Germans Trias i Pujol Hospital (HGTiP) in Badalona (Spain) that covers an area of approximately 700,000 inhabitants. After applying the exclusion criteria, 43 patients (male 30, female 13; median age 63 years, range 26–91) were included in our study. Most of the patients were initially treated according to the Spanish CETLAM protocols, based on the use of idarubicin or fludarabine with cytarabine. A total of 12 out of 43 patients underwent allogeneic stem cell transplant (ASCT) following chemotherapy. The patient’s risk (favorable, intermediate, adverse) was evaluated according to ELN 2017 recommendations, based on cytogenetic and/or molecular abnormalities [16]. Details of patient population and statistics are provided in Table 1 and Table 2 respectively. All patients enrolled in this study provided their informed consent in accordance with the Declaration of Helsinki. All procedures were in accordance with the internal protocols of our laboratory, which were authorized by the HGTiP Clinical Investigation Ethical Committee, in agreement with current Spanish legislation.

### Flow cytometry methods

All EDTA-anticoagulated bone marrow (*n* = 38) and blood (*n* = 5) samples were prepared using a modified previously developed method [27] aimed at avoiding the lysis of erythrocytes during sample preparation, which can result in unwanted damage to leukocytes, and conceivably to leukemic cells. As most enzyme functions are performed at 37°C, we first established an optimal Alkaline Phosphatase Live Stain (APLS, Thermo Fisher Scientific) stable loading time (t = 20 min) and temperature (T = 37°C) to measure ALP enzymatic activity [14]. This dye is a cell-permeable fluorescent substrate for ALP that is non-toxic to cells. Importantly, ammonium chloride- and paraformaldehyde-based lysing solutions impair and almost completely abrogate ALP staining, are not recommended for detection of ALP+ cells (Supplementary Figure 1). The KG-1a cell line, derived from Human Caucasian bone marrow acute myelogenous leukemia [31], was used as a positive control for highly expressing ALP+ cells (Supplementary Figure 2).

Briefly, our staining strategy used Vybrant™ DyeCycle™ Violet stain (DCV, Thermo Fisher Scientific), a low cytotoxicity permeable DNA-specific dye that can be used for DNA content cell cycle and stem cell Side Population analysis by flow cytometry [32]. DCV can be excited with violet 405 nm laser light and can be used for simultaneous measurement with APLS, which is excited with blue 488 nm laser light and its emission can be collected using a standard FITC filter (for example 530/30 nm). Subsequently, cells were stained with fluorophore-conjugated monoclonal antibodies for 20 min at room temperature as recommended by the manufacturer (PE-CD123, PECy5-CD34 and PECy7-CD117, Sysmex GmbH). The improved phycoerythrin (PE) signal using 561 nm excitation together with the fact that there is no need for color compensation between FITC and PE under 488 nm and 561 nm excitation, results in the improved immunophenotypic analysis of leukemic cells by multicolor flow cytometry [33].

Stained cells were diluted with Hank’s Balanced Salt Solution (HBSS) (1000 µL final volume) prior to sample acquisition. All cell measurements were done using the Attune™ Acoustic Focusing Cytometer and the Attune™ NxT Acoustic Focusing Cytometer (Thermo Fisher). Samples were acquired at 25–100 µL/min sample rates, and a minimum of 100,000 DCV+ events were collected per sample when possible. Threshold levels were set empirically using a Violet-Side Scatter (SSC) vs. DCV-H dual parameter plot to eliminate debris and the large numbers of red blood cells that are found in unlysed blood or bone marrow from detection. DCV was excited at 405 nm and its emission was collected using the following filter combination: 413 LP, 495 DLP, and 440/50 BP in the VL2 detector.

**Figure 4:**
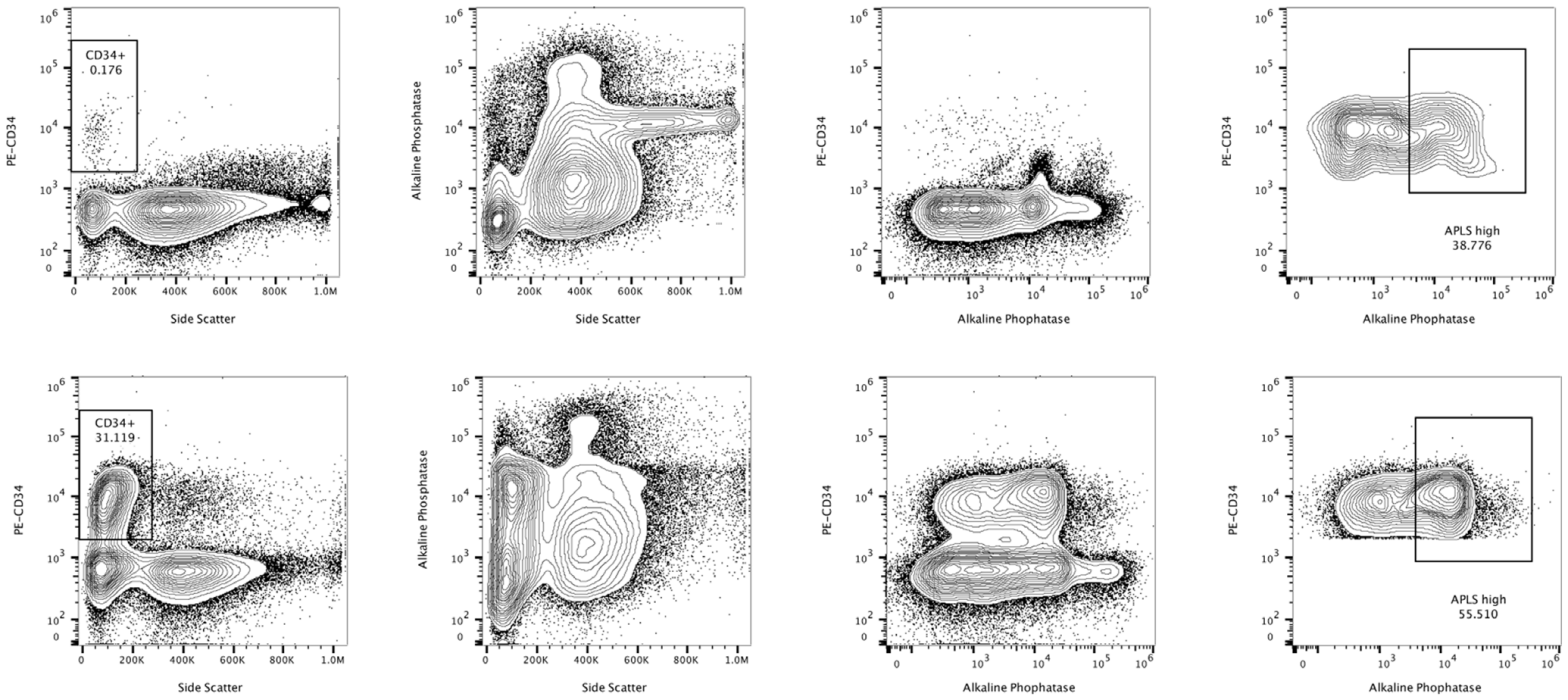
Representative flow cytometric study of the alkaline phosphatase activity in a patient of the ALP ≥ 12% group. Alkaline phosphatase positive cells are represented in combination with CD34 staining at diagnosis (upper row) and after relapse (lower row). Reference contour plots for two bone marrow aspirates are compared in the same patient, displaying high levels of alkaline phosphatase activity in combination with CD34 staining. The statistics in the region represents percentage of the gate.

SSC was detected using the violet laser 405 nm with a 405/10 nm bandpass filter. APLS was detected with the blue laser 488 nm excitation and a 530/30 nm bandpass filter in the BL1 (Blue Laser) detector. For the Attune™ NxT upgraded with the yellow laser kit, PE was excited at 561 nm and its emission was collected using the following filter combination: 595 LP, 600 DLP, and 585/16 BP in the YL1 detector. PECy5 was detected with the yellow laser 561 nm excitation and a 695/40 nm bandpass filter in the YL3 detector. PECy7 was detected with the yellow laser 561 nm excitation and a 780/60 nm bandpass filter in the YL4 detector. APLS, PE, PECy5, PECy7 and DCV fluorescence are displayed on a logarithmic scale. Representative contour plot analysis for two bone marrow aspirates at diagnosis and relapse, used to calculate ALP+ cell numbers in combination with CD34 staining is shown in Figure 4.

ALP+ cell numbers were determined in parallel to flow cytometry immunophenotyping of leukemia cells performed at the HGTiP clinical hematology laboratory. Standard immunophenotyping was performed on a FC500 (Beckman Coulter) flow cytometer until October 2016, and on a Navios™ flow cytometer (Beckman Coulter) from this date onwards. Blast cells were identified using cell surface protein markers according to the ELN recommendations [16]. MPO/CD45/CD34/CD117/CD123/Myeloid/T-Lymphoid/B-lymphoid and megakaryocyte (MK) markers were used for typifying the blastic population through the identification of LAIPs (whenever possible), or through the detection of abnormalities in the different-from-normal cellular distribution in association with a low expression of CD45 and low SSC. A minimum of 20,000 events were studied at diagnosis. A marker was considered as positive when was expressed by ≥20% of blastic cells. Assessment of MRD was performed on bone marrow aspirates with a minimum sensitivity of 0.1%. LAIPs identified at diagnosis were carefully looked for, and shifts in the relative scatter distribution of the different-from-normal pattern were also taken into account.

### Statistical methods

We used receiver operating characteristic (ROC) analysis, area under the curve (AUC), sensitivity and specificity to measure prognostic accuracy of our test in predicting EFS. The optimal cut-off value was identified according to the Youden’s Index to classify patients into two groups [34]. Differences between categorical variables in each group were compared using two-sided Fisher’s exact tests. Differences between continuous variables in each group were compared using two-sided Wilcoxon rank sum tests. EFS and OS rates were estimated by the method described by Kaplan and Meier [35]. EFS was defined as the time from diagnosis until progression, death, or the last follow-up date. OS was defined as the time from diagnosis until death or the last follow-up date. Patients that underwent ASCT were censored. Differences in survival between each group were tested for statistical significance using the two-sided log-rank test with the Bonferroni method to adjust *P* value. Univariate analysis using the Cox proportional hazards model was performed to investigate the impact of % ALP at diagnosis on EFS and OS, adjusting for the following variables: age, sex, ELN prognostic-risk group, blast percentage, white blood cell (WBC) count and AML type (*de novo* vs, secondary). Factors prognostic for EFS and OS with a *P*-value < 0.5 in the univariate analysis were studied in a multivariate analysis. A *P*-value < 0.05 was considered statistically significant. R Studio version 1.1.463 (https://www.R-project.org/) was used for all statistical analysis.

## CONCLUSIONS

In this study we have found that increased cellular alkaline phosphatase activity in leukemic cells at diagnosis was significantly associated with a higher risk of relapse, or treatment resistance, and mortality. Due to the small number of patients enrolled in this study, this finding requires further investigation.

## SUPPLEMENTARY MATERIALS




