# Supplementary material for: Flow cytometric significance of cellular alkaline phosphatase activity in acute myeloid leukemia
Source: Oncotarget. 2019 Dec 10;10(65):6969–80. doi: 10.18632/oncotarget.27356 (PMC6916750; doi:10.18632/oncotarget.27356)
Supplement: Supplementary file 1 [file oncotarget-10-6969-s001.pdf]

Flow cytometric significance of cellular alkaline phosphatase activity in acute myeloid leukemia

SUPPLEMENTARY MATERIALS

As most enzyme functions are performed at 37°C, we first established an optimal Alkaline Phosphatase Live Stain (APLS, Thermo Fisher Scientific) stable loading time (t = 20 min) and temperature (T = 37°C) to measure ALP enzymatic activity. This dye is a cell-permeable fluorescent substrate for ALP that is non-toxic to cells. Importantly, ammonium chloride- and paraformaldehyde-based lysing solutions can impair and almost completely abrogate ALP staining, are not recommended for detection of ALP+ cells. The KG-1a cell line, derived from Human Caucasian bone marrow acute myelogenous leukemia, were used as a positive control for highly expressing ALP+ cells.

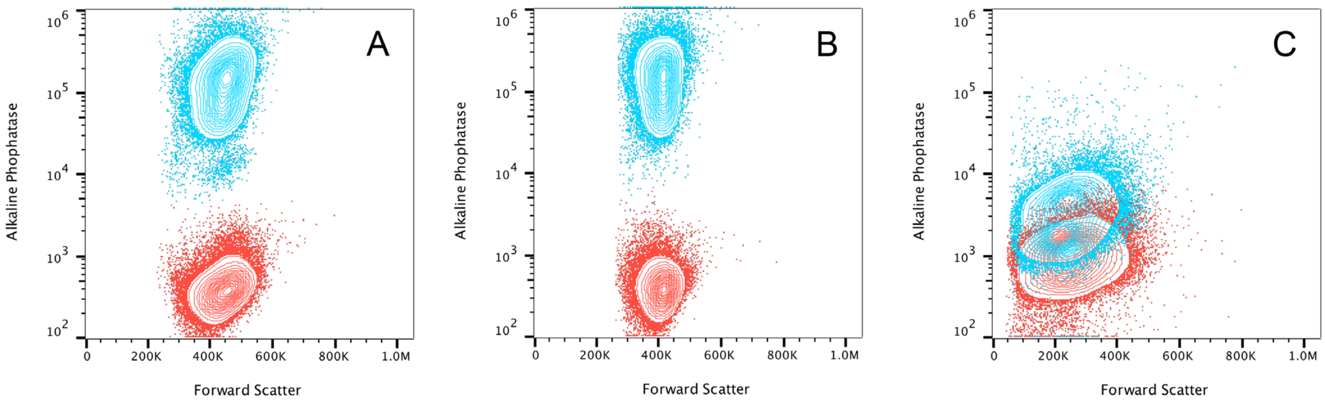

Supplementary Figure 1: Effect of lysing agents on ALP expression by flow cytometry. This figure shows the use of KG1a cells to separate positive and negative ALP cases. In terms of ALP expression measurements, control data are used to demonstrate that the measurements are reproducible. KG1a cells were prepared with the no-lyse nowash approach (**A**), with ammonium chloride (**B**) and with a paraformaldehyde based solutions (**C**). Ammonium chloride based solution supports lysis of erythrocytes, it is buffered and optimized for gentle lysis of erythrocytes, with minimal effect on leukocytes. However, erythrocyte lysing with agents such as ammonium chloride is often ineffective and can damage cells and cell function. Note that KG1a cells in (**B**) and (**C**) exhibit less forward scatter than the same cells in (**A**), due to some type of damage.

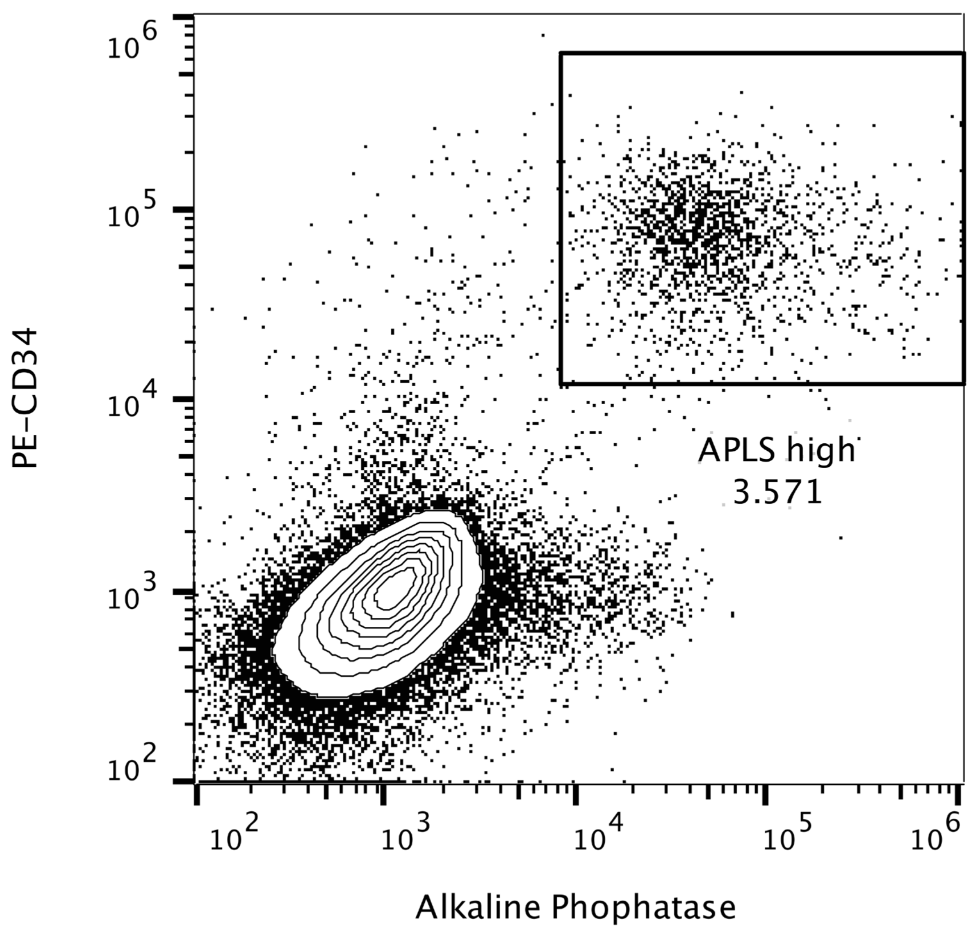

Supplementary Figure 2: ALP expression by flow cytometry. In terms of ALP expression measurements, control data are used to demonstrate that the measurements are reproducible. This figure shows the use of KG1a cells to separate positive and negative ALP cases. KG1a cells were manually spiked into samples of blood from a healthy donor to assess the numbers of CD34+/APLS+ cells.

Supplementary Table 1: Individual patient characteristics. See Supplementary Table 1
